# The Prognosis of Hepatocellular Carcinoma Treated with Sorafenib in Combination with TACE

**DOI:** 10.31557/APJCP.2020.21.6.1797

**Published:** 2020-06

**Authors:** Yusuke Kimura, Rena Kaneko, Yuichiro Yano, Kentaro Kamada, Takashi Ikehara, Hidenari Nagai, Yuzuru Sato, Yoshinori Igarashi

**Affiliations:** 1 *Department of Gastroenterology, Kanto Rosai Hospital 1-1 Kizukisumiyoshi-cho, Nakahara-ku, Kawasaki, Kanagawa, 211-8510 Japan. *; 2 *Division of Gastroenterology and Hepatology, Department of Internal Medicine (Omori), School of Medicine, Faculty of Medicine, Toho University 6-11-1 Omorinishi, Ota-ku, Tokyo, 143-8541 Japan. *

**Keywords:** Hepatocellular carcinoma, sorafenib, transcatheter chemoembolization, tyrosine kinase inhibitor

## Abstract

**Objective:**

Sorafenib have been shown to be effective in the treatment of advanced HCC and has been standard therapy since its release in Japan in 2009 (Llovet et al., 2008; Cheng et al., 2009). However, due to a low response rate, more aggressive combination treatment has been utilized as a multimodal strategy. The present study aimed to determine the efficacy of sorafenib alone and in combination with transarterial chemoembolization (TACE) for the treatment of advanced HCC.

**Methods:**

All patients with unresectable advanced HCC who were prescribed sorafenib at Kanto Rosai Hospital were included in the study. Five-year overall survival (OS) rates were estimated for patients treated with sorafenib alone or in combination with TACE. Multivariate and univariate regression analyses were performed to identify factors affecting OS. Analysis using propensity score matching and inverse-probability weights were also performed.

**Results:**

A total of 46 patients were treated with sorafenib up to June 2018. The total sorafenib dose administered was higher in the TACE combination group (70900 mg vs. 24000 mg vs. with sorafenib alone), although the relative dose intensity was lower (11.7% vs. 17.6%, respectively). The 5-year survival prognosis estimated using the Kaplan-Meier method was longer in patients treated with sorafenib in combination with TACE versus sorafenib alone (36.3% vs. 7.7%). Combination with TACE was the only factor associated with improved OS in both univariate and multivariate analysis. Among cases matched by propensity scores the hazard rate for combination with TACE was 0.067 (95% CI 0.091-1.128).

**Conclusion:**

With an array of therapeutic options currently available, it is important to determine the efficacy of different multimodal strategies, such as sorafenib combined TACE, for patients with unresectable HCC.

## Introduction

Sorafenib is an oral multikinase inhibitor that blocks tumor cell proliferation and represents the standard therapy for progressive hepatocellular carcinoma (HCC) after locoregional therapy for patients who have adequate liver function. However, the benefit of sorafenib monotherapy in real-world clinical practice has been modest with a low response rate and relatively frequent serious adverse effects. Therefore, sorafenib in combination with other treatment modalities had been expected to improve therapeutic efficacy.

Since its introduction to the market, sorafenib has been strictly indicated for HCC in patients with Barcelona Clinic Liver Cancer (BCLC) stage C or those with progressive disease after locoregional therapy with preserved liver function (Colagrande et al., 2015). 

Using such strict administration criteria, the SHARP trial (Llovet et al., 2008) and the Asia-Pacific trial (Cheng et al., 2009) revealed that sorafenib treatment prolonged median overall survival (OS) by only 3 months and was associated with a response rate (RR) of approximately 3%. To improve the efficiency of sorafenib treatment and prolong the prognosis of advanced HCC, multimodal treatment strategies have been employed, even though management guidelines for HCC in Japan still recommend using sorafenib as a monotherapy (Kudo et al., 2011; Hagihara et al., 2014; Ikeda et al., 2016; Ikuta et al., 2018). These studies used a continuous sorafenib administration protocol concurrently with transarterial chemoembolization (TACE), or an alternative additional treatment strategy involves sequential TACE with interruption of sorafenib treatment. Consequently, the use of subclassification criteria for treatment strategies is advocated in intermediate-stage HCC in Japan (Kudo et al., 2015). Sorafenib combined with TACE has been widely applied to treat unresectable HCC in clinical practice (Hu et al., 2014).

In our hospital, sorafenib had been administrated at a relatively early stage of HCC such as Kinki criteria B2, as suggested by Kudo and colleagues (Kudo et al., 2015), and a multimodal strategy has been used since sorafenib was approved for use. In the present study, we evaluated the efficacy of sorafenib treatment alone versus in combination with TACE. 

## Materials and Methods

All patients with unresectable advanced HCC who were prescribed sorafenib treatment between April 2009 and June 2018 at Kanto Rosai Hospital were included in the study. 

Cases receiving conventional TACE in combination with sorafenib were allocated to the TACE combination group. The decision to perform TACE was made at the discretion of the physician based on the presence of hypervascular tumors or aggressive growth of any part of multiple tumors. Sorafenib treatment was interrupted 4 days before TACE and was restarted 1-3 days after TACE. Dose reduction or interruption of sorafenib was performed according to general recommendations. The clinical course and 5-year survival rate of all patients were retrospectively analyzed. 

Chi-square test was performed to demonstrate differences in baseline characteristics. For continuous variables, Mann-Whitney U test was used to compare groups. The 5-year survival rate was estimated using the Kaplan-Meier method with right censoring at the 5-year mark. Cox proportional hazard models were used to calculate OS. 

Multivariate and univariate logistic regression analyses were conducted to identify factors related to RR. We adjusted for the following covariates: age, etiology, laboratory values (platelet count, alanine aminotransferase [ALT], total bilirubin, albumin, alpha-fetoprotein [AFP], protein induced by vitamin-K absence-II [PIVKA-II]), Child-Pugh grade, TNM stage (UICC), Barcelona Clinic Liver Cancer (BCLC) stage, and sorafenib treatment alone or combination with TACE. 

We also performed propensity score matching since the baseline characteristics may be influenced by patient selection for either sorafenib treatment alone or combination with TACE. The multivariable logistic regression model for propensity score matching included the following parameters: sorafenib treatment alone or combination with TACE, BCLC stage, TNM stage, serum AFP level, and etiology (viral vs. other). After the propensity score has been established, we applied 1:1 matching using the nearest-neighbor matching method with a 0.05 caliper width. The hazard rate (HR) for combination with TACE was estimated by Cox regression analysis. Owing to the small total number of cases and significant differences in the frequency of baseline BCLC stages, this would have resulted in very small numbers of patients in each group after propensity score matching. We also tried to estimate the average treatment effect (ATE) for survival time using inverse-probability weights (IPW) as treatment effects. The HR was estimated by Cox regression analysis using the same parameters as the propensity score matching model.

All p-values were 2-sided, and a p-value <0.05, <0.01 was considered statistically significant. All analyses were conducted using STATA/MP15.0 software (Stata Corp LP, College Station, TX, USA).

Informed consent was obtained from all patients before any study procedures were undertaken. This study conformed to the ethical guidelines of the Declaration of Helsinki and was approved by the ethics committee of the Japan Organization of Occupational Health and Safety Kanto Rosai Hospital (2014-34, 2018-11).

## Results

A total of 46 patients were prescribed sorafenib prescription in our hospital. Baseline characteristics of the patients, the response to treatment and subsequent therapies used in all cases are shown in Table 1. There were no significant differences in gender, age, etiology, or laboratory values between the sorafenib alone group and TACE combination group. The etiology of the group designated as “other” was speculated to be liver cirrhosis caused by non-alcoholic steatohepatitis (NASH), although a pathogenic diagnosis was not obtained in 8 cases. Performance status (PS) (based on Eastern Cooperative Oncology Group criteria) and BCLC stage were significantly better in the TACE combination group than in the sorafenib alone group.

The response and progression were evaluated by modified response evaluation criteria in solid tumors criteria (m-RECIST) (Lencioni and Llovet, 2010). The best response identified with m-RECIST in each case was summarized. In the TACE combination group, 3 cases (21.4%) showed a complete response (CR), 2 (14.3%) had a partial response (PR), and 6 (42.9%) had stable disease (SD). 

Both the response rate (RR) and disease control rate (DCR) were higher in the TACE combination group (35.7% and 78.6%, respectively) compared with the sorafenib alone group (3.1% and 37.5%, respectively). The sorafenib prescription was permanently discontinued in 35 cases up to April 2019, and the reasons for termination were PD (including death) for 30 cases and adverse events for 5 cases. After termination of sorafenib, 10 cases received subsequent therapies: i.e., regorafenib in 3 cases (6.5%) and lenvatinib in 7 cases (15.2%). Thirty-six cases underwent best supportive care. Roughly 21 patients (45.7%) started sorafenib at 200 mg/day prescription dose according to their age, body weight, and the physician’s discretion. If the initial dose was tolerated, the dose was increased to 400 mg, 600 mg, or 800 mg/day. The median relative dose intensity (RDI) was 15.6% in all cases, 17.6% in the sorafenib alone group, and 11.7% in the TACE combination group.

Adverse events were determined by Common Terminology Criteria for Adverse Events (CTCAE) version 5.0 are presented. Diarrhea (19.6%), hand-foot Syndrome (31.1%), hoarseness (19.6%), and alopecia (26.1%) were mainly detected but were tolerable with strict management of the sorafenib dose in all but 5 cases including 3 cases with drug induced liver failure.

**Figure 1:**
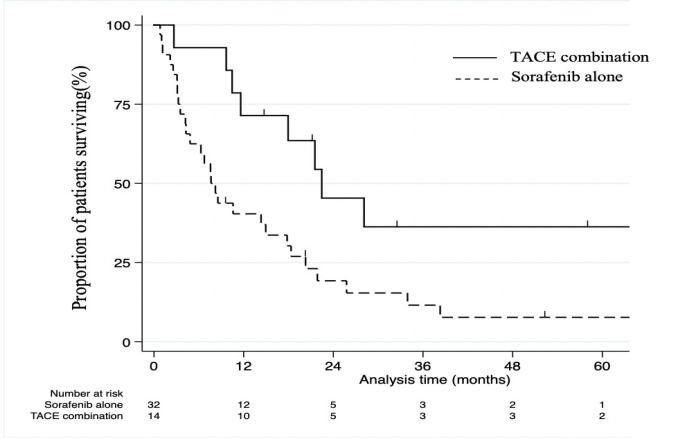
Kaplan-Meier Curves for Overall Survival Treated with Sorafenib Alone and Combination with TACE. Overall survival was estimated using the Kaplan-Meier method for sorafenib alone and combination with TACE patients with right censoring at the 5-year mark. P-values were calculated from log-rank tests. The survival rate was significantly higher in the TACE combination group versus the sorafenib alone group

Figure 1 shows the 5-year survival rates for all cases who were prescribed sorafenib. Kaplan-Meier survival curves for OS for sorafenib alone or in combination with TACE are shown. The survival rate was 15.6% in all cases. The survival rate was longer in combined with TACE (36.3%) versus sorafenib alone (7.7%; p=0.01). The Median Survival time (MST) of sorafenib alone group was 8.0 months compared to 21.5 months in combination with TACE group.

Multivariate and univariate logistic regression analyses for factors affecting RR were performed (Table 2). Multivariate analysis showed that combination with TACE was independently associated with obtaining an RR, while univariate analysis showed that only combination with TACE was associated with an RR.

**Figure 2:**
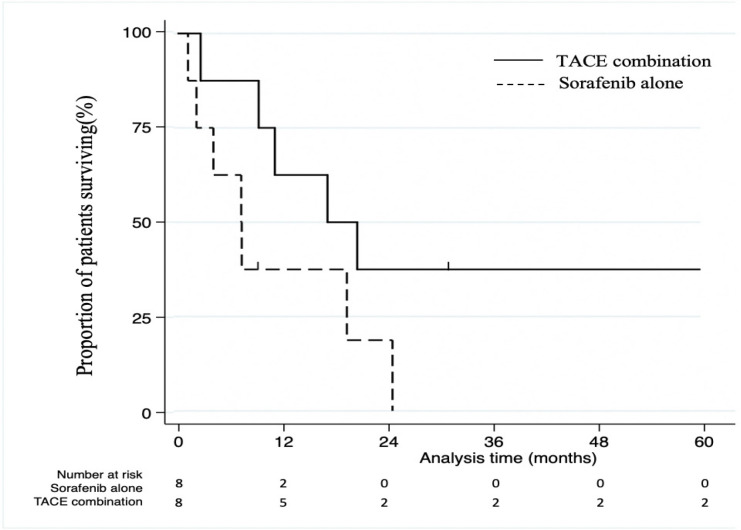
Kaplan-Meier Curves for Overall Survival after Propensity Score Matching. Survival was estimated using the Kaplan-Meier method for propensity score matched patients with right censoring at the 5-year mark. P-values were calculated from log-rank tests. The survival rate was not significantly higher in the TACE combination group versus the sorafenib alone group, even though median survival time was 7.4 months in sorafenib alone group compared to 22.3 months in combination with TACE group

Table 3 shows baseline characteristics after propensity score matching. Sixteen patients after 1:1 matching (8 patients each in the sorafenib alone group and combination with TACE group, respectively) were identified. For matched cases, the 5-year survival rate was 30.0% in the combination with TACE group versus 0% in the sorafenib alone group; log-rank test showed no statistically significant difference (Figure 2). MST was 7.4 months in sorafenib alone group compared to 22.3 months in combination with TACE group. The HR estimated by Cox regression model was 0.067 (95% CI 0.091-1.128) for the TACE combination group and the ATE was 14.0 months. Using IPW, the HR was 0.048 (95% CI 0.091-0.982) for the TACE combination group and the ATE was 12.7 months.

**Table 1:** Baseline Characteristics and Course of Treatment

Characteristics, N(%)	All	Sorafenib alone	TACE combination	*P*-value^1^
Overall (N=46)				
Gender				
Male (%)	37 (80.4)	27 (84.4)	10 (71.4)	0.308
Female (%)	9 (19.6)	5 (15.6)	4 (28.6)	
Age				
Age at prescription median (IQR)^2^	72.4 (68.0 : 78.4)	73.5 (65.8 : 78.1)	71.8 (69.6 : 78.8)	0.519
Etiology				
HCV	17	11 (34.4)	6 (42.9)	0.583
HBV	16	12 (37.6)	4 (28.6)	0.559
Alcoholic	4	2 (6.3)	2 (14.3)	0.373
Other	9	7 (21.8)	2 (14.3)	0.55
Laboratory values				
Platelet count ×10^4^/μL median (IQR)^2^	12.3 (8.8 : 16.9)	12.5 (9.1 : 16.8)	11.3 (7.6 : 16.9)	0.72
ALT IU/L median(IQR)^2^	34 (19 : 49)	33 (19 : 47)	39 (24 : 56)	0.607
Total bilirubin IU/L median(IQR)^2^	0.9 (0.7 : 1.4)	0.9 (0.7 : 1.4)	0.9 (0.6 : 1.2)	0.321
Albumin IU/L median (IQR)^2^	3.7 (3.4 : 4.1)	3.6 (3.4 : 4.1)	3.8 (3.5 : 4.2)	0.241
AFP ng/ml median (IQR)^2^	33.7 (8.3 : 1490)	83 (9.9 : 2973)	7.8 (22.5 : 138)	0.321
PIVKA-II ng/ml median (IQR)^2^	171.5 (26 : 519.5)	819 (28 : 7147)	111 (23: 223)	0.084
Chid-Pugh grade				
A (%)	45 (98)	36 (97)	9 (100)	
B (%)	1 (2)	1 (3)	0 (0)	
C (%)	0 (0)	0 (0)	0 (0)	
Stage (UICC)				
IVA (%)	35 (76.1)	23 (71.8)	12 (85.7)	0.311
IVB (%)	11 (23.9)	9 (28.1)	2 (14.3)	
Performance status				
0 (%)	25 (54.3)	13 (40.6)	12 (85.7)	0.005^a^
1-2 (%)	21 (45.6)	19 (59.4)	2 (14.3)	
BCLC Stage				
B (%)	19 (41.3)	9 (28.1)	10 (71.4)	0.006^a^
C (%)	27 (58.7)	23 (62.2)	4 (28.6)	
Response to treatment (mRECIST), N(%)				0.017^a^
Complete response	4 (8.7)	1 (3.1)	3 (21.4)	
Partial response	13 (28.2)	11 (34.4)	2 (14.3)	
Stable disease	23 (50.0)	17 (53.1)	6 (42.9)	
Progressive disease	6 (13.0)	3 (9.4)	3 (21.4)	
Response rate, N(%)	6 (13.0)	1 (3.1)	5 (35.7)	0.003^b^
Desease control rate, N(%)	23 (50.0)	12 (37.5)	11 (78.6)	0.01^a^
Subsequent treatment, N(%)				
Change to regorafenib	3 (6.5)	3 (9.4)	0 (0)	
Change to lenvatinib	7 (15.2)	4 (12.5)	3 (21.4)	
Discontinued permanently	36 (78.0)	25 (78.1)	11 (78.6)	
Number of tablets at start N(%)				
200 mg	21 (45.7)	14 (43.8)	7 (50.0)	0.215
400 mg	19 (41.3)	12 (37.5)	7 (50.0)	
800 mg	6 (13.0)	6 (18.8)	0 (0)	
Sorafenib dose (mg)				
Median (IQR)2	32500 (12400:102200)	24000 (8500:85100)	70900 (19600:180400)	0.044^a^
Relative dose intensity (%) (IQR)^2^	15.6 (5.6:28)	17.6 (7.7:26.7)	11.7 (5.2:29.7)	0.535

^1^, P-values <0.05^a^ or <0.01^b^ were considered to be statistically significan; ^2^, IQR:Interquartile range

**Table 2:** Regression Models for Response Cases

Variables	Multivariate regression		Univariate regression	
	Hazards ratio (95%CI)	*P*-value^1^	Hazards ratio (95%CI)	*P*-value^1^
Age at prescription (y)	0.48 (-0.012-0.019)	0.638	0.46 (-0.010-0.017)	0.648
Etiology	-0.42 (0.135-0.089)	0.678	0.13 (-0.086-0.985)	0.894
Laboratory values				
Platelet count (×10^4^/μL)	0.94 (-0.001-0.002)	0.352	1.11 (-0.006-0.02)	0.274
ALT (IU/L)	-1.07 (-0.005-0.002)	0.239	-1.20 (-0.004-0.010)	0.236
Total bilirubin (IU/L)	0.58 (-0.093-0.167)	0.564	-0.47 (-0.125-0.077)	0.639
Albumin (IU/L)	0.78 (-0.125-0.282)	0.439	1.36 (-0.058-0.294)	0.182
AFP (ng/ml)	-0.48 (-0.005-0.033)	0.632	-0.80 (-0.004-0.002)	0.430
PIVKA-II (ng/ml)	-0.36 (0.009-0.066)	0.723	-0.47 (-0.009-0.055)	0.642
Chid-Pugh grade	0.14 (-1.431-1.642)	0.890	-0.38 (-0.834-0.567)	0.703
TNM Stage	0.635 (-0.372-0.230)	0.635	-0.44 (-0.291-1.874)	0.664
BCLC Stage	0.93 (-0.159-0.424)	0.362	-0.45 (-0.254-0.161)	0.678
Combination with TAE	2.66 (0.079-0.598)	0.012^a^	3.30 (0.127-0.525)	0.002^b^

^1^, *P*-values <0.05^a^ or <0.01^b^ were considered to be statistically significant.

**Table 3.** Baseline Characteristics of Patients after Propensity Score Matching

Characteristics, N(%)	All	Sorafenib alone	TACE combination	*P*-value
Overall (N=16)				
Gender				
Male (%)	11 (68.7)	6 (75.0)	5 (62.5)	0.59
Female (%)	5 (31.3)	2 (25.0)	3 (37.5)	
Age				
Age at prescription median (IQR)^1^	77.0 (70.0 : 79.3)	77.0 (72.0 : 78.4)	75.3 (69.9 : 75.3)	0.836
Etiology				
Viral	11 (68.7)	5 (63.5)	6 (75.0)	0.59
Other	5 (31.3)	3 (37.5)	2 (35.0)	
Laboratory values				
Platelet count ×10^4^/μL median (IQR)^1^	8.5 (6.3 : 13.1)	7.5 (5.4 : 11.1)	10.8 (6.9 : 13.3)	0.401
ALT IU/L median(IQR)^1^	44 (32 : 59)	45 (34 : 71)	43 (27 : 54)	0.528
Total bilirubin IU/L median(IQR)^1^	1.2 (0.9 : 1.4)	1.3 (0.9 : 1.4)	1.1 (0.7 : 1.6)	0.561
Albumin IU/L median (IQR)^1^	3.9 (3.5 : 4.2)	4.0 (3.6 : 4.2)	3.8 (3.3 : 4.3)	0.674
AFP ng/ml median (IQR)^1^	31 (7.8 : 9399)	10.8 (7.2 : 89872)	65 (8.8 : 7613)	0.998
PIVKA-II ng/ml median (IQR)^1^	67 (21 : 431)	67 (28 : 46330)	66 (19: 181)	0.385
Chid-Pugh grade				
A (%)	16 (100)	8 (100)	8 (100)	
Stage (UICC)				
IVA (%)	11 (68.7)	6 (75.0)	5 (82.5)	0.59
IVB (%)	5 (31.3)	2 (25.0)	3 (37.5)	
Performance status				
0 (%)	12 (75.0)	6 (75.0)	6 (75.0)	1.000
1-2 (%)	4 (25.0)	2 (25.0)	2 (25.0)	
BCLC Stage				
B (%)	11 (68.7)	6 (75.0)	5 (82.5)	0.59
C (%)	5 (31.3)	2 (25.0)	3 (37.5)	

^1^IQR, Interquartile range

## Discussion

Several clinical trials in advanced-stage HCC patients have examined the effect of sorafenib in combination with other modalities including TACE (Kudo et al., 2011; Chao et al., 2015), hepatic arterial infusion chemotherapy (HAIC) (Hagihara et al., 2014; Ikuta et al., 2018), chemotherapy (Petrini et al., 2012; Abou-Alfa et al., 2019) and other molecular targeting agents (Zhu et al., 2015). While these studies tended to show a favorable prognosis, there are still no strict recommendations regarding multimodal treatment strategies (Marrero et al., 2018). 

In the present study, using all cases, sorafenib in combination with TACE was effective and superior to treatment with sorafenib alone. Combination with TACE was the only factor associated with improved OS in both univariate and multivariate analyses. Though statistical significance disappeared after propensity scores were matched, survival curves showed a clearly good prognosis and the MST was improved in combination with TACE. There were only 16 matched cases, representing 34% of the total number of participants, and thus the statistical power for the analysis was low. 

The possible mechanisms for long surviving in combination with TACE group might be summarized into three points.

First, we have to mention to the mechanism of tumor angiogenesis. The results of the START trial suggest that sorafenib enhances the efficacy of TACE (Chao et al., 2015). The length of treatment with sorafenib was greater in patients who received combined therapy, which could have resulted from the better response rate and longer survival times (Choi et al., 2013). This finding suggests that TACE and sorafenib have additive benefits on treatment outcomes; i.e., a tumor debulking effect of TACE combined with relief of ischemia due to the angiogenic action of sorafenib (Li et al., 2013; Liang et al., 2013a). There is a consensus that TACE induces ischemic or hypoxic changes that result in increased vascular endothelial growth factor (VEGF) activity in surviving cancer tissue (Llovet et al., 2008). On the other hand, sorafenib targets several core processes in tumor development and progression. Specifically it is able to inhibit tyrosine kinases of the VEGF signaling pathway to reduce tumor angiogenesis and RAF kinase, associated with an inhibition of the MAPK/ERK pathway leading to reduced cell proliferation (Llovet et al., 2008). It also leads to mitochondrial dysfunction and decreased NAD and adenosine triphosphate (ATP) levels that regulate critical cellular processes necessary for cancer cell growth (Garten et al., 2019). Thus, the use of a potent multikinase inhibitor, such as sorafenib, with TACE may limit the proliferative, proangiogenic behavior of tumor with suppression the surge of proangiogenic factors after TACE (Chao et al., 2015; Kudo et al., 2019). Accordingly, the decrease of post-TACE angiogenesis by combined sorafenib strategy might cause prolong interval between each TACE and result in good efficacy of TACE through normalization of tumor vessels (Chao et al., 2015; Kudo et al., 2019).

Second, we can argue from the point of MicroRNA (miRNA) in cancer (Lee et al., 1993). It is reported that miRNAs can regulate more than 60% of the protein-coding genes in cells (Lee et al., 1993). The relationship between decreased miRNA expression and the increased expression of the oncogenic target genes became evident, suggesting a tumor-suppressor function for miRNAs (Johnson et al., 2005). Concerning about HCC, the abnormal regulation of miRNAs had been reported and several studies suggested the involvement of miRNAs in multi-drug resistance link to the poor prognosis (Pratama et al., 2019). It has been reported that high serum miR-181a-5p levels are accompanied with OS in patients with BCLC-C HCC (Nishida et al., 2017). In performed TACE cases, miR-200a level is an independent prognostic factor associated with HCC outcome (Liu et al., 2014). MicroRNA-221 correlates poor outcome in patients with sorafenib (Kim et al., 2017; Jin et al., 2018). It was also said the long-tern exposure to sorafenib leads to epithelial-mesenchymal transition (van Malenstein et al., 2013), and the dysregulation of HIF-1 was associated with sorafenib resistance, known to elicit the hypoxic environment by enhancing angiogenesis (Liang et al., 2013b; Xu et al., 2014).

In summary, miRNAs play a significant role in targeting specific genes involved in different but interpolated pathways: thus, TACE and sorafenib might influence to the prognosis of HCC through various complexed abnormal regulations of miRNAs (Pratama et al., 2019).

Thirdly, from the point of immune system and cytokine production must be under consideration. It was reported, sorafenib administration induces Th1 dominance and prevent tumor cells from escaping the host immune system and improve the host immune response to cancer (Nagai et al., 2012; Nagai et al., 2015). Administration of sorafenib in HCV patients may change Th1 dominance to Th2 dominance (Nagai et al., 2012). 

Natural killer (NK) cells are cytotoxic and are known to be effective for various types of tumor cells (Tonn et al., 2001). Because NK cells account for 25-50% of the total number of liver lymphocytes, its dysfunction in HCC related to the poor prognosis in HCC patient (Sun et al., 2015). The number of NK cells in the peripheral blood is significantly positively correlated with survival rates and the prognosis of liver cancer (Hoechst et al., 2009; Chew et al., 2012). It is reported that the cellular immune function of HCC patients is significantly impaired by TACE, while the peripheral blood NK cell was not changed significantly (Lu et al., 2002). On the other hand, sorafenib could affect the proportions and functions of peripheral CD56brightCD16- and CD56dimCD16+ NKcells, which was associated with OS (Lu et al., 2002). Some studies also reported that sorafenib might influence the activity by modulating NK cells (Cao et al., 2011; Sprinzl et al., 2013). In addition, the case presentation of complete response of HCC with sorafenib showed her peripheral NK cell activity was quite high (Kim et al., 2018). 

Hence, anti-HCC treatment strategies in combination with multimodal strategies might be effective through NK cells functions (Sun et al., 2015). 

Some potential limitation might exist in this study. First, because it was conducted at only 1 hospital, the total number of cases was too small. Because of this limitation, comparisons among the sorafenib alone group and TACE combination group were not stratified for baseline characteristics and multivariate analysis such as logistic regression or Cox proportional hazard models may not be reliable. To address this limitation, propensity score matching was performed, but almost half the cases were lost as a result. Furthermore, despite propensity score matching, the subset of patients not treated with TACE and surely worse clinical presentation so as to make them not eligible to TACE. Therefore, there is also the possibility that the improved survival is related to the possibility to use two different treatments in the same patient and this depends upon both disease presentation and general status of the patient. Our conclusions need to be verified in a larger cohort of patients. Second, because TACE was performed as the discretion of the physician, there are limitations regarding the generalizability of the results such that these tumors might have been more amenable to TACE. However, this study provides important knowledge about advanced HCC treatment with sorafenib.

In conclusion, a good prognosis was demonstrated in patients who received long-term sorafenib therapy in combination with TACE. Use of a suitable multimodal strategy for HCC at a relatively early stage may help to improve the efficacy of therapy.

## Statement Conflicts of Interest

None.
